# Catastrophic health expenditures incurred by families in 2003, 2009 and 2018 in the Federal District, Brazil: evolution and composition

**DOI:** 10.1590/S2237-96222024v33e20231358.en

**Published:** 2025-01-27

**Authors:** Pedro Henrique Alves Santos, Theo da Fonseca Torres, Letícia Xander Russo, Everton Nunes da Silva

**Affiliations:** 1 Universidade de Brasília, Programa de Pós-Graduação em Ciências e Tecnologias em Saúde, Brasília, DF, Brazil; 2 Universidade de Brasília, Programa de Pós-Graduação em Economia, Brasília, DF, Brasil; 3 Universidade Federal da Grande Dourados, Faculdade de Administração, Ciencias Contábeis e Economia, Dourados, MS, Brasil; 4 Universidade de Brasília, Faculdade de Ceilândia, Brasília, DF, Brasil

**Keywords:** Gasto Catastrófico en Salud;, Gastos en Salud, Costos de los Medicamentos, Economía y Organizaciones para la Atención de la Salud, Estudios de Series Temporales, Catastrophic Health Expenditure, Health Expenditure, Drug Costs, Health Care Economics and Organizations, Time Series Studies

## Abstract

**Objective:**

To investigate the evolution of prevalence of catastrophic health expenditure in the Brazilian Federal District at three different times (2003, 2009 and 2018), as well, to identify the composition of outof- pocket expenditure in the respective years.

**Method:**

Time series study, using descriptive data from the Family Budget Survey. Prevalence was stratified by consumption quintiles.

**Results:**

754 households were selected as a sample in 2003, 695 in 2009 and 1,000 in 2018. Taking a 10% consumption threshold, prevalence of catastrophic expenditure was 12.3% (95%CI 9.6;14.9) in 2003, 15.3% (95%CI 12.1;18.3) in 2009 and 14.1% (95CI% 11.8;16.2) in 2018. Households with lower income had higher prevalence of catastrophic expenditure. Medicines have a greater burden on expenditure of low-income families.

**Conclusions:**

There was an increase in prevalence of catastrophic expenditure in the Federal District. Medicines were the main expense for the poorest families.

## Study contributions

**Main results:**

Prevalence of catastrophic health expenditure increased in the Federal District, rising from 12.3% in 2003 to 15.3% in 2018, reaching its peak in 2009 (14.1%) (10% threshold), whereby medication was the main health expenditure in the poorest strata.

**Implications for services:**

There is room for improving the performance of health services, expanding their health care provision strategies from the viewpoint of equity, through intersectoral policies. Special attention must be given to pharmaceutical assistance.

**Perspectives:**

The Federative Unit governments have a fundamental role in achieving financial protection for health care in Brazil, so that the sum of subnational efforts results in the achievement of target 3.8 of the UN Sustainable Development Goals.

## RESUMEN

**Objective:**

Investigar la evolución de prevalencia del gasto catastrófico en Distrito Federal en tres momentos diferentes (2003, 2009 y 2018), así como identificar la composición del gasto directo en salud de los recursos de las familias en los respectivos años.

**Method:**

Estudio de series temporales, utilizando datos descriptivos de Encuesta de Presupuestos Familiares. La prevalencia se estratificó por quintiles de consumo.

**Results:**

Se seleccionaron como muestra 754 hogares en 2003, 695 en 2009 y 1.000 en 2018. El umbral del 10% del consumo, la prevalencia del gasto catastrófico fue 12,3% (IC95% 9,6;14,9) en 2003, del 15,3% (IC95% 12,1;18,3) en 2009 y del 14,1% (IC95% 11,8;16,2) en 2018. Hogares pobres tuvieron una mayor prevalencia de gastos catastróficos. Medicamentos tienen un mayor peso en los gastos de familias pobres.

**Conclusions:**

Hubo un aumento en la prevalencia de gastos catastróficos en el Distrito Federal. Los medicamentos fueron el principal gasto en familias paupérrimas.

## INTRODUCTION

Out-of-pocket payment for health care using families’ own financial resources is the most unfair funding regime, as it conditions the use of health care to individuals’ ability to pay, potentially generating inequities in access to health.^1^ It is also associated with greater risk of incurring impoverishing or catastrophic expenditure.^2,3^ In 2015, financial risk protection regarding health care became an integral part of the Sustainable Development Goals (target 3.8), ratified by the 193 United Nations members. Globally, several initiatives have been carried out to monitor financial risk in health care, mostly measured through the occurrence of catastrophic expenditure.^4^ There was a growth in the population that incurred catastrophic expenditure worldwide(out-of-pocket health expenditure exceeding 10% of the family budget); in turn, the number of people affected rose from 12.7% of the world population in 2015 (940 million people) to 13.2% (996 million) in 2017, mainly concentrated in low- and middle-income countries and in the Asia-Pacific region.^5^

Brazil has national estimates on the percentage of families that incurred catastrophic health expenditure, varying between 17.9%^6^ and 33.4%,^7^ depending on the way the percentage was calculated (10% in relation to income or household consumption, respectively). However, estimates for subnational entities are scarce. It is important to monitor the performance of Brazilian Federative Units regarding financial protection of health care, since achieving the national target is a result of subnational efforts. Furthermore, monitoring catastrophic expenditure can also be used as a diagnosis of health services, when there is a high proportion of out-of-pocket expenditure by families on a certain health product or service. For example, families accounted for 87.7% of spending on medications in Brazil in 2019.^8^ In this sense, there is room for improvements in pharmaceutical assistance in the Brazilian National Health System (*Sistema Único de Saúde - SUS*), aimed at providing access to medications in a timely manner and based on rational use.^9^

We chose to investigate the evolution of prevalence of catastrophic expenditure in the Federal District, because it is the Federative Unit with the highest monthly household per capita income in Brazil (R$ 2,913.00).^10^ Despite this, socioeconomic inequalities persist in the District that is home to the country’s capital. In 2020, the Federal District Social Vulnerability Index (*Índice de Vulnerabilidade Social do Distrito Federal* - IVS-DF) was 0.34 on a scale that varies from 0 to 1, in which higher values represent greater social vulnerability. Although the IVS-DF is considered average, there are large disparities between the District’s administrative regions, with the following distribution: very low (14 administrative regions), low (11), medium (3), high (1) and very high (3).^11^ This asymmetric pattern can significantly affect the prevalence of catastrophic expenditure in the Federal District. Additionally, the Federal District has some particularities in relation to other Federative Units, such as joint action, encompassing both state and municipal functions, as well as centralized management, given the absence of autonomous municipal bodies.^12^

Monitoring prevalence of catastrophic health expenditure presupposes monitoring this indicator in the short and long term. Lack of standardization of score estimates compromises such monitoring, thus making it difficult for policy makers to address it. Only one study was identified that measured catastrophic health expenditure in the Federal District, and that was only for the year 2018. Progress is still needed to standardize methods, empirical strategies and data sources, in order to allow for more accurate monitoring of financial protection of health care, even more so at the subnational level. One of the main contributions of this study is its providing methodological, empirical and data source uniformity over different periods, in addition to producing more comparable estimates in terms of the evolution of prevalence of catastrophic expenditure.

The objective of this study was to investigate the evolution of prevalence of catastrophic health expenditure in the Brazilian Federal District at three different times (2003, 2009 and 2018), as well, to identify the composition of family out-of-pocket expenditure on health in the respective years. 

## METHODS

### Study design 

This is a time series study, with a descriptive approach, using secondary data from the Family Budget Survey (*Pesquisa sobre Orçamentos Familiares* - POF), covering the periods 2002/2003, 2008/2009 and 2017/2018. The data was accessed via the website of the Brazilian Institute of Geography and Statistics (*Instituto Brasileiro de Geografia e Estatística* - IBGE), whereby we downloaded the files on March 7, 2023. As such, we sought to verify possible inequalities related to prevalence of catastrophic expenditure by consumption bracket. 

### Study context 

The Federal District is home to the capital of Brazil, with a territorial area of 5,760.784 km^2^, a resident population of 2,817,068 people and population density of 489.01 inhabitants per km^2^. The Federal District’s health system organization is complex compared to other Brazilian Federative Units, with entities with different legal personalities, purposes and service provision to the population.^13^ Furthermore, the Federal District has a constitutional fund, a financial transfer from the Union (Federal Administration) that covers several sectors, including health, as it hosts the country’s capital. In the past, this amount covered health expenditure, but over the years, and due to rapid demographic transition, the resources transferred have become insufficient to balance the books.^12^

### Participants

We used data from the last three editions of the Family Budget Survey (2002/2003, 2008/2009 and 2017/2018). The Family Budget Survey is carried out by IBGE focusing on family consumption structures, making it possible to outline a profile of the population’s living conditions based on examining household budgets. The Family Budget Survey unit of analysis is the household, selected through probabilistic sampling, providing national, macro-region and Federative Unit data.^14^ We used the sampling weights used by each edition of the Family Budget Survey. The Family Budget Survey includes several questionnaires, called *dossiers*. In order to generate the results of this study, data were extracted from the following dossiers for each of the three versions of the survey: i) individual expenditure, ii) residents, iii) living conditions, iv) collective dossier, v) household, vi) other income and vii ) inventory. For the 2002-2003 and 2008-2009 Family Budget Surveys, data from dossiers relating to 90-day expenditure, domestic services and 12-month expenditure were also used; for the 2008-2009 and 2017-2018 Family Budget Surveys, estimated rent was included; and for the 2017-2018 Family Budget Survey, collective expenditure and health expenditure limitations were included. As the Family Budget Survey includes all family members, there is no age cutoff as an exclusion criterion. However, the head of the family is an adult (age greater than or equal to 18 years old).

### Variables and data sources

The variable of interest was family catastrophic health expenditure, measured through out-of-pocket health expenditure (numerator) that exceeds a certain percentage of total income or total family consumption (denominator). The percentages most used in the literature are 10%, 25% and 40%,^1,15^ and these we also adopted in this study. We opted for consumption rather than income, as it is more sensitive to the ability to make out-of-pocket payments for health care, particularly when expenditure on basic needs, such as housing and food, are deducted.^16^ The equation is expressed by dividing out-of-pocket health expenses (numerator) by family consumption expenditure (denominator), multiplied by 100, to obtain the percentage. For the 10% and 25% thresholds, total household consumption was taken as the denominator of the equation; in turn, for the 40% threshold, expenditure on rent and food was subtracted from total family consumption.

The composition of out-of-pocket health expenditure (numerator) included medications and health products, hospitalizations, consultations and examinations/tests, excluding voluntary payments with health insurance. This is a World Health Organization and World Bank recommendation, according to which any reimbursement from a third party (public or private system) should be excluded.^16^

In order to verify which medications had the greatest impact on household expenditure, they were classified according to the Anatomical Therapeutic Chemical – (ATC) classification system, in which active substances are divided into different groups, according to the body organ or body system on which they act and their therapeutic, pharmacological and chemical properties.^17^ Additionally, the percentage of households that had limitations in purchasing medications was calculated, with data from the health limitation dossier, available only for the 2017-2018 Family Budget Survey. 

### Statistical analysis

The data were analyzed descriptively. To this end, we calculated the percentage of households that incurred catastrophic health expenditure at three points in time (2003, 2009 and 2018), taking different thresholds into account (10%, 25% and 40%). In order to analyze socioeconomic inequalities in catastrophic health expenditure, households were divided into five equal parts (quintiles), stratified by income and consumption. The sampling weights of the three Family Budget Surveys were taken into consideration. The analyses were performed using Stata 14.0. 

The amounts in Brazilian Real (BRL) for the comparison between the years were updated according to the Broad National Consumer Price Index (*Índice Nacional de Preços ao Consumidor Amplo*), with the amounts being adjusted to August 2023 price levels,^18^ that being the last month of the index available at the time of conducting the study. In order to convert the amounts from the Family Budget Surveys into current amounts, we used the reference date of the surveys as made available by IBGE. The reference month for converting values was January of the last year of each Family Budget Survey (2003, 2009 and 2018).^14,19,20^ We also performed a descriptive analysis of the composition of out-of-pocket health expenditure according to its destination, including expenditure on medications and health products, hospitalizations, consultations and examinations/tests, and health insurance. Emphasis was placed on out-of-pocket expenditure on medications, adding expenditure data classified according to self-reported medications. 

### Ethical aspects 

As this study was comprised of analysis based on secondary research sources, it did not need to be submitted to an Ethics and Research Committee, in accordance with National Health Council Resolution No. 466. 

## RESULTS

**Figure 1:**
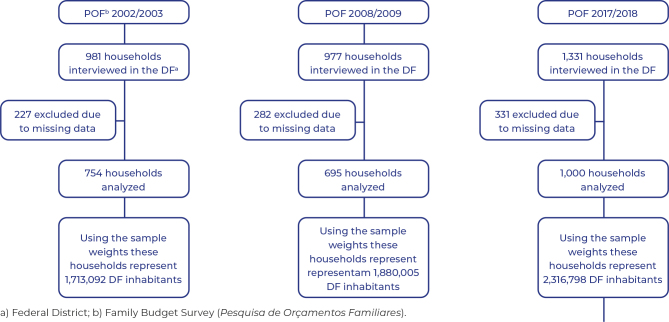
Flowchart of households included in the analysis of three representative national surveys of household consumption, 2002/2003, 2008/2009 and 2017/2018, Federal District, Brazil

As part of the overall Family Budget Survey, the following number of households were selected for the survey specifically in the Federal District: 1,214 households in 2002/2003, 1,703 in 2008/2009 and 1,850 in 2017/2018. Of these, in 2002/2003, 981 households were interviewed (80.8% of the selected households); in 2008/2009, 977 households were interviewed (57.3% of selected households); and in 2017/2018, 1,331 households were interviewed (71.9% of selected households). After exclusion due to missing data, the sample selected for the study was 754 households analyzed in the 2002/2023 Family Budget Survey (76.8% of households interviewed), 695 households analyzed in the 2008/2009 Family Budget Survey (71.1% of households interviewed) and 1,000 households in the 2017/2018 Family Budget Survey (75.1% of households interviewed). Using the sampling weights, 1,713,092 Federal District inhabitants were represented in the 2002/2003 Family Budget Survey, 1,880,005 in the 2008/2009 Family Budget Survey and 2,316,798 in the 2017/2018 Family Budget Survey (Figure 1).

Using the three thresholds for calculating prevalence of catastrophic expenditure, there was an increase in prevalence between 2003 and 2009, followed by a drop between 2009 and 2018. However, when considering 2003 as the base period, an increase in prevalence of catastrophic expenditure was identified in relation to 2018, with the exception of the 2017/2018 Family Budget Survey, considering the threshold of 40% or more of total consumption, less food and rent (2.7% [95%CI 1.4;3.8] versus 2.5% [95%CI 1.5;3.4], respectively) (Table 1). 

**Table 1:** Prevalence of catastrophic health expenditure (%) with confidence intervals 95% (95%CI), by consumption quintile for selected consumption thresholds (10%, 25% and 40%), stratified by years of the Family Budget Survey, Federal District, Brazil

Consumption quintile	Families committing 10% or more of total consumption			Families committing 25% or more of total consumption			Families committing 40% or more of total consumption, less food and rent		
	POF ^a^ 2002/2003 % (IC95%)	POF 2008/2009 % (IC95%)	POF 2017/2018 % (IC95%)	POF ^a^ 2002/2003 % (IC95%)	POF 2008/2009 % (IC95%)	POF 2017/2018 % (IC95%)	POF ^a^ 2002/2003 % (IC95%)	POF 2008/2009 % (IC95%)	POF 2017/2018 % (IC95%)
1	12.5 (6.3;18.6)	12.1 (5.9;18.1)	21.4 (15.5;27.2)	3.9 (0.2;7.5)	2.4 (-0.6;5.4)	4.2 (1.3;7.1)	5.3 (1.4;9.2)	4.8 (0.8;8.6)	5.5 (2.2;8.7)
2	11.6 (6.2;17.0)	14.0 (7.3;20.6)	16.7 (11.2;22.2)	1.5 (-0.1;3.1)	4.5 (0.1;8.9)	2.0 (0.1;3.9)	2.1 (0.3;3.9)	6.3 (1.2;11.3)	2.0 (-0.1;4.0)
3	11.3 (5.6;17.0)	13.4 (7.0;19.6)	11.7 (7..1;16.2)	1.6 (-0.4;3.6)	3.3 (0.1;6.44)	2.3 (-0.1;4.6)	1.5 (-0.5;3.4)	3.2 (0.1;6.3)	1.2 (-0.3;2.7)
4	13.3 (6.7;19.8)	19.3 (11.7;26.7)	12.3 (7.5;17.0)	1.4 (-0.6;3.3)	5.9 (1.5;10.2)	1.7 (-0.1;3.3)	2.5 (0.1;4.9)	4.8 (0.7;8.8)	2.2 (0.2;4.1)
5	12.8 (6.8;18.6)	15.8 (9.06;22.54)	10.3 (6.2;14.2)	2.5 (-0.2;5.1)	2.6 (-0.4;5.5)	3.3 (0.9;5.6)	1.7 (-0.6;4.0)	2.0 (-0.8;4.8)	2.1 (0.2;3.9)
DF	12.3 (9.6;14.9)	15.3 (12.1;18.3)	14.1 (11.8;16.2)	2.1 (1.0;3.2)	3.8 (2.1;5.5)	2,7 (1.6;3.7)	2,7 (1.4;3.8)	4.1 (2.3;5.8)	2.5 (1.5;3.4)

a) Family Budget Survey.

Considering the distribution of household consumption and the threshold of 10% or more of consumption as an indicator of catastrophic expenditure, we found that households with the lowest consumption (quintile 1) had an increase of 71.2% in prevalence of catastrophic expenditure between 2003 and 2018, while households with the highest consumption (quintile 5) showed a 19.5% decrease in prevalence in the same period. In turn, taking the 25% and 40% thresholds, the lower and upper tails of the distribution (quintiles 1 and 5) showed an increase in prevalence of catastrophic expenditure, which was more pronounced in households with the highest consumption (25% threshold: quintile 1 = 7.6% versus quintile 5 = 32.0%; 40% threshold quintile 1 = 3.7% versus quintile 5 = 23.5%) (Table 1). 

Table 2 shows that the composition of out-of-pocket health expenditure differed in relation to household income, with a predominance of expenditure on medications in households with the lowest income (quintile 1); and expenditure on health insurance, in households with the highest income (quintile 5). Furthermore, hospitalization expenses were close to zero in all income quintiles. We did not find a well-defined pattern of percentage of expenditure on consultations and examinations/tests in relation to income quintiles, although the two tails of the distribution have very different percentages (quintile 1 = 5.3% versus quintile 5 = 16.6%, in the 2017/2018 Family Budget Survey).

**Table 2:** Distribution of out-of-pocket health expenditure (%) with confidence intervals 95% (95%CI), by income quintile, stratified by type of expense and by year of the Family Budget Survey, Federal District, Brazil

**Type of out-of-pocket health expenditure**	**Year of the POF** ^b^	**Income quintile**					
		**1 (↓ income) % (IC95%)**	**2 % (IC95%)**	**3 % (IC95%)**	**4 % (IC95%)**	**5 (↑ income) % (IC95%)**	**DF** ^a^ **% (IC95%)**
Medication	2002/2003	86.5 (80.7;92.3)	71.4 (63.7;78.9)	57.3 (49.5;65.0)	42.7 (36.1;49.2)	26.4 (21.0;31.7)	56.8 (53.4;60.1)
	2008/2009	83.4 (76.7;90.0)	79.2 (71.6;86.7)	82.8 (76.6;88.9)	65.6 (57.5;73.6)	60.1 (52.8;67.3)	72.3 (68.8;75.7)
	2017/2018	88.3 (81.5;95.0)	77.9 (70.7;84.9)	73.5 (66.8;80.1)	66.0 (59.9;72.0)	41.7 (38.4;44.8)	54.7 (52.1;57.3)
Hospitalization	2002/2003	- (-)	- (-)	- (-)	- (-)	0.4 (-0.3;1.0)	0.1 (-0,1;0.2)
	2008/2009	- (-)	- (-)	- (-)	- (-)	- (-)	- (-)
	2017/2018	- (-)	- (-)	- (-)	- (-)	0.1 (-0.1;0.2)	0.1 (-0.1;0.2)
Consultation and examination	2002/2003	12.2 (6.7;17.7)	25.2 (17.7;32.6)	21.0 (14.8;27.0)	27.3 (21.2;33.3)	28.7 (22.3;34.9)	23.0 (20.1;25.9)
	2008/2009	12.1 (6.1;18.1)	16.1 (9.3;22.7)	10.7 (6.0;15.2)	14.7 (9.6;19.8)	15.7 (10.2;21.2)	14.1 (11.5;16.6)
	2017/2018	5.3 (1.4;9.1)	17.4 (10.9;23.7)	16.9 (11.5;22.1)	18.4 (13.8;22.9)	16.6 (13.5;19.7)	16.4 (14.2;18.5)
Health insurance	2002/2003	1.2 (0.1;2.3)	3.4 (0.5;6.2)	21.7 (14.2;29.1)	30.0 (23.2;36.7)	44.6 (38.2;50.9)	20.1 (17.5;22.7)
	2008/2009	4.5 (1.1;7.8)	4.7 (1.2;8.3)	6.5 (2.2;10.8)	19.7 (11.9;27.4)	24.1 (17.6;30.5)	13.6 (10.8;16.4)
	2017/2018	6.4 (1.2;11.5)	4.8 (1.0;8.5)	9.7 (4.6;14.7)	15.6 (10.5;20.6)	41.6 (37.9;45.3)	28.8 (26.1;31.5)

a) Federal District; b) Family Budget Survey.

With regard to the average amounts spent on medications in households in the Federal District, adjusted for inflation in the period, there was an increase in spending between 2003 and 2009 (45.0%), followed by a small drop between 2009 and 2018 (-0.5%). A similar pattern was found when considering the lower and upper tails of the income distribution, with spending growth being more pronounced in the poorest households, between 2003 and 2009 (quintile 1 = 67.3% versus quintile 5 = 30.5%) , and the drop in spending in the wealthiest households (quintile 1 = -9.2% versus quintile = -14.4%) was greater between 2009 and 2018 (Table 3).

**Table 3:** Distribution of out-of-pocket expenditure on medication by income quintile, stratified by the Anatomical Therapeutic Chemical (ATC) Classification and by year of the Family Budget Survey, Federal District, Brazil

**ATC** ^a^ **Classification**	**Year of the POF** ^c^	**Income quintile in (BRL)**					
		**1 (↓ income)**	**2**	**3**	**4**	**5 (↑ income)**	**DF** ^b^
Alimentary tract and metabolism (A)	2002/2003	1.90	5.29	8.72	25.73	25.18	12.80
	2008/2009	23.67	28.07	22.73	16.45	54.03	30.53
	2017/2018	11.13	15.28	25.10	28.47	54.67	29.19
Cardiovascular system (C)	2002/2003	4.53	5.14	11.56	51.03	50.55	23.39
	2008/2009	17.11	20.12	50.89	56.62	93.89	51.93
	2017/2018	9.36	21.37	25.97	39.70	43.29	29.53
Dermatologicals (D)	2002/2003	2.31	5.06	4.30	4.00	12.17	5.35
	2008/2009	0.40	1.31	0.06	0.56	9.35	2.83
	2017/2018	0.45	3.74	1.29	6.27	13.04	5.62
Genito urinary system and sex hormones (G)	2002/2003	2.68	7.02	1.83	1.94	7.95	4.26
	2008/2009	1.54	3.13	6.50	10.25	3.62	5.14
	2017/2018	1.30	4.18	4.67	6.90	3.38	4.12
Antiinfective for systemic use (J)	2002/2003	2.20	11.39	20.52	22.06	15.06	13.92
	2008/2009	2.69	3.17	2.27	8.39	8.46	5.40
	2017/2018	4.52	6.85	4.77	14.54	12.89	9.19
Antineoplastic and immunomodulating agents (L)	2002/2003	9.17	9.52	19.75	17.01	28.94	16.24
	2008/2009	5.51	18.61	7.59	16.68	33.63	17.91
	2017/2018	3.56	4.70	14.90	9.86	11.77	9.26
Nervous system (N)	2002/2003	4.59	3.49	3.54	21.76	25.72	11.30
	2008/2009	3.35	5.01	27.54	12.83	31.04	17.18
	2017/2018	7.57	25.19	16.91	52.53	24.69	26.11
Antiparasitic products. insecticides and repellants (P)	2002/2003	0.49	0.47	0.01	-	0.13	0.23
	2008/2009	0.07	0.01	-	0.26	-	0.07
	2017/2018	0.38	1.09	-	0.39	0.41	0.39
Respiratory system (R)	2002/2003	4.00	6.37	10.24	12.94	9.57	8.47
	2008/2009	2.63	12.37	13.05	13.06	16.00	11.99
	2017/2018	5.95	6.76	8.99	11.54	14.97	10.12
Sensory organs (S)	2002/2003	0.36	0.48	7.08	5.51	14.30	5.10
	2008/2009	0.03	0.30	-	1.51	1.01	0.64
	2017/2018	2.65	2.72	3.88	5.49	9.62	5.26
Various (V)	2002/2003	12.20	10.20	19.79	20.40	31.87	18.24
	2008/2009	17.34	17.44	19.93	46.59	37.99	29.39
	2017/2018	20.57	35.69	42.43	51.15	58.51	43.34
Average Total	2002/2003	44.43	64.43	107.34	182.38	221.44	119.30
	2008/2009	74.34	109.54	150.56	183.20	289.02	173.01
	2017/2018	67.44	127.57	148.91	226.84	247.24	172.13

a) Anatomical Therapeutic Chemical Classification; b) Federal District; c) Family Budget Survey.

Table 3 also shows which medications accounted for the largest proportion of out-of-pocket expenditure on them in households in the Federal District. There was a predominance of medications related to classifications V (various), C (cardiovascular system) and A (alimentary tract and metabolism), representing 45.6% of total spending on medications in 2003, 64.6% in 2009, and 59.2% in 2018. No substantial changes were identified in the composition of medications according to income bracket.

Table 4 shows that low-income families had greater limitation in purchasing medications in all ATC classifications. Medications related to the nervous system (N) were those with the smallest difference between the extremes of income, with the percentage of purchase limitation being 1.5 times higher in quintile 1 than in quintile 5 (2.2% versus 1.4%, respectively). The alimentary tract and metabolism classification (A) showed the greatest difference in the percentage of purchasing limitation, being 11.5 times higher in quintile 1 compared to quintile 5.

**Table 4:** Percentage of households with limitations for purchasing medications by income quintile, stratified by the Anatomical Therapeutic Chemical (ATC) Classification, Federal District, Brazil, POF 2017/2018

**Type of medication with limitation (ATC** ^a^ **classification)**	**Year of the POF** ^c^	**Income quintile %**					
		**1 (↓ income)**	**2**	**3**	**4**	**5 (↑ income)**	**DF** ^b^
Alimentary tract and metabolism (A)	2017/2018	4.6	0.9	1.7	3.6	0.4	2.1
Cardiovascular system (C)	2017/2018	5.3	4.4	4.6	5.6	2.1	4.2
Dermatologicals (D)	2017/2018	1.8	-	0.9	1.2	1.0	1.0
Genito urinary system and sex hormones (G)	2017/2018	0.4	0.6	-	0.7	0.2	0.4
Antiinfective for systemic use (J)	2017/2018	1.7	-	1.2	0.5	0.2	0.7
Antineoplastic and e immunomodulating agents (L)	2017/2018	2.7	1.3	0.8	0.7	1.3	1.3
Nervous system (N)	2017/2018	2.2	1.3	1.3	1.5	1.4	1.5
Antiparasitic products. insecticides and repellants (P)	2017/2018	0.4	-	-	-	-	-
Respiratory system (R)	2017/2018	3.2	0.3	2.0	-	-	1.0
Sensory organs (S)	2017/2018	0.7	0.4	-	0.3	0.3	0.3
Various (V)	2017/2018	12.5	10.7	6.8	6.0	1.8	7.1

a) Classificação Anatômica-Terapêutico-Química; b) Distrito Federal; c) Pesquisa de Orçamentos Familiares.

## DISCUSSION

In this study, we were able to monitor prevalence of catastrophic expenditure in the Federal District at three points in time. In general, the peak prevalence of catastrophic expenditure occurred in 2009, regardless of how it was measured (10%, 25% or 40% threshold). Furthermore, households with the lowest consumption (quintile 1) tended to spend proportionally more on health care than households with the highest consumption (quintile 5). In other words, households with lower income tended to have higher prevalence of catastrophic health expenditure. It is also noteworthy that medications have a large impact on the out-of-pocket health expenditure of low-income families, representing more than 83% of such expenditure. Medications that led to highest family expenditure were those related to the alimentary tract and metabolism (A), cardiovascular system (C) and various (V) classifications. These classifications were also those that had the greatest limitation as to their purchase. 

When considering the 10% consumption threshold, prevalence of catastrophic health expenditure ranged between 12.3% (2003), 15.3% (2009) and 14.1% (2018) for households in the Federal District. These estimates were lower than those found by Araújo and Coelho (2021) for the Federal District (28.7%).^7^ Although both studies used the same database (2017/2018 Family Budget Survey), the use of different methodological strategies may explain these differences. In the study by Araújo and Coelho (2021),^7^ which made estimates for Brazil as a whole and for its Federative Units, no mention is made about the use of the survey sample weights, the way consumption was calculated (denominator) or the number of households included in the calculation. Other studies have provided estimates of prevalence of catastrophic health expenditure at a national level (analyzing Brazil as a whole), covering the periods from 1987 to 2003, 2002 and 2003, and 2002 to 2009, however, they were not stratified by Federative Unit.²¹^,^²²^,^²³

In turn, at the subnational level, we found two studies, one covering the state of Minas Gerais,^24^ with estimates between the years 2009 and 2013, and the other covering the municipality of Porto Alegre,^25^ using data for the period from July to September 2003. In the city of Porto Alegre,^25^ the study was conducted using data with a representative sample of families served by the Family Health Strategy between July and September 2003. The study used the 5%, 10% and 20% family income thresholds and a 40% threshold for the household’s ability to pay, in order to calculate the proportion of households that incurred catastrophic expenditure in Porto Alegre. In the case of the 10% threshold, prevalence of 28.8% of families was found. In addition to being conducted in two different Federative Units, there are also methodological differences between the two studies – for example, the definition of the threshold denominator, which, in our study, was consumption rather than income.^25^ The study conducted in the state of Minas Gerais,^24^ assessed catastrophic health expenditure between 2009, 2011 and 2013, using the Minas Gerais Household Sample Survey (*Pesquisa por Amostra de Domicílios de Minas Gerais*). That study used 10% and 25% household income thresholds to measure prevalence of catastrophic health expenditure. Taking the 10% threshold, from 2009 to 2011, there was a 22.5% increase in the proportion of catastrophic expenditure, and from 2011 to 2013, a 13.5% decrease. Taking the 25% threshold, from 2009 to 2011, there was an increase of 20.3%, and a decrease of 24.4% from 2011 to 2013.^24^ This same pattern was found in our study for prevalence of catastrophic expenditure, using 10%, 25% and 40% thresholds, for consumption.

The inverse relationship between income/consumption and catastrophic spending has also been reported in other Brazilian publications, all of which analyzed Brazil as a whole, with periods ranging from 1987 to 2009,^7,21,23^ corroborating the findings of our study. At a global level, this inverse relationship was also found between the variables in question in Bangladesh, in 2010,^26^ in South Korea, between 2009 and 2012,^27^ and in European Union member countries, between 2000 and 2017.^5^

Out-of-pocket expenditure on medications as the most important component for the poorest households has also been found in other studies. At a national level, based on the 2017-2018 Family Budget Survey,^7^ spending on medications was higher among the group of poorest families (decile 1), representing 84% of this group’s health expenditure, unlike the group of wealthiest people (decile 10), in which it only accounted for 29%. In Minas Gerais,^24^ expenditure on medications, in the three years analyzed (2009, 2011 and 2013), accounted for approximately 94% of out-of-pocket expenditure. In 2003, in Porto Alegre,^25^ using data from households covered by the Family Health Strategy, the main expenditure on health in the group of poorest households (quintile 1) was on medications, BRL 20.07 on average (at 2003 price levels, or BRL 45.10 at August 2023 price levels after adjustment for inflation), while in the group of wealthiest households the main expense was on health insurance, BRL 138.90 on average (at 2003 price levels, or BRL 312.12 at August 2023 price levels after adjustment for inflation). 

Within the SUS, pharmaceutical assistance has shown significant progress in normative terms, examples of which are the creation of the National Medication Policy (*Política Nacional de Medicamentos*) (1998), the National Pharmaceutical Assistance Policy (*Política Nacional de Assistência Farmacêutica*) (2004) and the Pharmacy Program for the Brazilian People (*Programa Farmácia Popular do Brasil*) (2004).^28^ Despite this progress, problems with access to medications still persist, particularly for low-income people, as shown in our study, in which more than 83% of family health expenditure was allocated to buying medications in quintile 1 (lowest income households). When disaggregating expenditure on medications according to therapeutic purpose, classifications that include diabetes (A) and hypertension (C) predominate, with these medications being partially dispensed in primary care. According to data from the National Survey on Access, Use and Promotion of Rational Use of Medications (*Pesquisa Nacional sobre Acesso, Utilização e Promoção do Uso Racional de Medicamentos* - PNAUM), 25.7% (95%CI 23.4;28.2) and 21.1% (95%CI 18.1;24.4 ) of medications used to treat hypertension^29^ and diabetes^30^ were paid for by families (out-of-pocket expenditure), respectively.

This study contributes to the literature by systematizing evidence on the prevalence of catastrophic expenditure in the Brazilian Federal District, considering data from measurements taken three times over a period of 15 years. Furthermore, it provides unprecedented data on the breakdown of expenditure on medications according to therapeutic purpose and signals the occurrence of limitations for purchasing medications by income quintile. It is noteworthy that the data source used in this study is aligned with international recommendations on the topic,^1^ including probabilistic sampling and detailed data on consumption and income. Finally, the results of this study allow monitoring of financial protection/(lack of protection) of health care in the light of catastrophic expenditure, at the subnational level. 

Despite the strengths of this study, some limitations should be mentioned. First, the public Family Budget Survey data does not allow the microdata to be disaggregated by administrative region of the Federal District, which limited our analysis of the disparities that exist between its administrative regions (geographical aspect). In order to overcome this fact, we performed analyses by income quintiles, in order to capture asymmetries in prevalence of catastrophic expenditure by socioeconomic profile. People with lower consumption and/or lower income do indeed tend to be more affected by catastrophic expenditure and limitations as to buying medications. Secondly, over the last three editions of the Family Budget Survey, changes have occurred in some health-related consumption items; however, these changes were limited to a few items, with reduced impact on research comparability. Thirdly, because the Family Budget Survey sample for the Federal District was relatively small, with missing data, and the prevalence of catastrophic expenditure was low (especially at the 25% and 40% thresholds), it was not possible to estimate the factors associated with catastrophic expenditure. As a consequence, relationships between non-significant variables could be presented in a spurious way. The low number of observations also increases uncertainty about the real prevalence rate of catastrophic expenditure in the Federal District. We included confidence intervals in order to address this situation. 

Finally, we conclude that there was an increase in lack of financial protection of health care among families in the Federal District. Prevalence of catastrophic health expenditure increased in the Federal District, rising by 1.8% from 2003 to 2018, reaching its peak in 2009 (increasing by 3% from 2003 to 2009), taking occurrence of catastrophic expenditure as being expenditure equal to or greater than 10% of household consumption. As a recommendation for health services, there is room for expanding their operations with a focus on equity, especially in relation to access to medications for diseases such as diabetes and hypertension. This can effectively contribute to increasing financial protection of the health care of families with lower income, given that buying medications is the largest component of out-of-pocket expenditure for these familie.
