# Supplementary material for: Catastrophic health expenditures incurred by families in 2003, 2009 and 2018 in the Federal District, Brazil: evolution and composition
Source: Epidemiol Serv Saude. 2025 Jan 27;33:e20231358. doi: 10.1590/S2237-96222024v33e20231358.en (PMC11776070; doi:10.1590/S2237-96222024v33e20231358.en)
Supplement: Translation

# Translation: Gastos catastróficos em saúde incorridos pelas famílias nos anos de 2003, 2009 e 2018 no Distrito Federal: evolução e composição

**DOI:** 10.1590/S2237-96222024v33e20231358.pt

**Authors:** Pedro Henrique Alves Santos, Theo da Fonseca Torres, Letícia Xander Russo, Everton Nunes da Silva

**Affiliations:** 1 Universidade de Brasília, Programa de Pós-Graduação em Ciências e Tecnologias em Saúde, Brasília, DF, Brasil; 2 Universidade de Brasília, Programa de Pós-Graduação em Economia, Brasília, DF, Brasil; 3 Universidade Federal da Grande Dourados, Faculdade de Administração, Ciencias Contábeis e Economia, Dourados, MS, Brasil; 4 Universidade de Brasília, Faculdade de Ceilândia, Brasília, DF, Brasil

**Keywords:** Gasto Catastrófico em Saúde, Gastos em Saúde, Gasto com Medicamentos, Economia e Organizações de Saúde, Estudos de Séries Temporais

## Abstract

**Objetivo:**

Investigar a evolução da prevalência de gastos catastróficos no Distrito Federal em três momentos distintos (2003, 2009 e 2018), bem como identificar a composição dos gastos diretos em saúde dos recursos financeiros das famílias nos respectivos anos.

**Métodos:**

Estudo de série temporal, usando dados da Pesquisa sobre Orçamento Familiar. A prevalência foi estratificada por quintis do consumo.

**Resultados:**

Foram selecionados como amostra 754 domicílios em 2003, 695 em 2009 e 1.000 em 2018. Para o limiar de 10% do consumo, a prevalência dos gastos catastróficos foi de 12,3% (IC_95%_ 9,6;14,9) em 2003, 15,3% (IC_95%_ 12,1;18,3) em 2009 e 14,1% (IC_95%_ 11,8;16,2) em 2018. Domicílios com menor renda apresentaram maior prevalência de gastos catastróficos. Medicamentos têm maior peso no gasto das famílias de baixa renda.

**Conclusão:**

Houve aumento da prevalência de gastos catastróficos no Distrito Federal. Medicamentos foram o principal gasto das famílias mais pobres.

## Contribuições do estudo

**Principais resultados:**

A prevalência de gastos catastróficos na saúde aumentou no Distrito Federal, passando de 12,3%, em 2003, para 15,3% em 2018, atingindo seu pico em 2009 (14,1%) (limiar de 10%), sendo o medicamento o principal gasto em saúde nos estratos mais pobres.

**Implicações para os serviços:**

Há espaço para o aprimoramento da atuação dos serviços de saúde, ampliando suas estratégias de provisão de cuidados em saúde sob a lente da equidade, por meio de políticas intersetoriais. Especial atenção deve ser dada à assistência farmacêutica.

**Perspectivas:**

As unidades federativas têm um papel fundamental na obtenção da proteção financeira em saúde no Brasil, a fim de que o somatório dos esforços subnacionais resulte no atingimento da meta 3.8 dos Objetivos do Desenvolvimento Sustentável da ONU.

## INTRODUÇÃO

Na saúde, o pagamento direto proveniente dos recursos financeiros das famílias é o regime de financiamento mais injusto, pois condiciona a utilização dos cuidados de saúde à capacidade de pagamento dos indivíduos, podendo gerar iniquidades no acesso à saúde.^1^ Também é associado ao maior risco de se incorrer em gastos empobrecedores ou catastróficos.^2,3^ Em 2015, a proteção ao risco financeiro em saúde tornou-se parte integrante dos Objetivos do Desenvolvimento Sustentável (meta 3.8), ratificados pelos 193 signatários da Organização das Nações Unidas. Globalmente, várias iniciativas têm sido conduzidas para monitorar o risco financeiro em saúde, majoritariamente mensurado via ocorrência de gastos catastróficos.^4^ Houve um crescimento da população que incorreu em gastos catastróficos no mundo (gastos diretos em saúde superiores a 10% do orçamento familiar); por sua vez, a quantidade de pessoas atingidas passou de 12,7% da população mundial em 2015 (940 milhões de pessoas) para 13,2% (996 milhões) em 2017, concentrando-se principalmente em países de baixa e média renda e na região da Ásia-Pacífico.^5^

O Brasil dispõe de estimativas nacionais sobre o percentual das famílias que incorreram em gastos catastróficos em saúde, variando entre 17,9%^6^ e 33,4%,^7^ a depender da forma como o percentual foi calculado (10% em relação à renda ou ao consumo das famílias, respectivamente). No entanto, são escassas as estimativas para os entes subnacionais. É importante monitorar o desempenho das unidades federativas (UFs) brasileiras sobre a proteção financeira em saúde, visto que o atingimento da meta nacional é decorrência dos esforços subnacionais. Ademais, o monitoramento dos gastos catastróficos também pode ser usado como diagnóstico para os serviços de saúde, quando se verifica alta proporção do dispêndio direto das famílias com determinado produto ou serviço de saúde. Por exemplo, as famílias foram responsáveis por 87,7% do gasto com medicamentos no Brasil em 2019.^8^ Nesse sentido, há espaço para aprimoramentos da assistência farmacêutica no Sistema Único de Saúde (SUS), na direção do provimento de acesso aos medicamentos em tempo oportuno e baseado no uso racional.^9^

Optou-se por investigar a evolução da prevalência dos gastos catastróficos no Distrito Federal, porque é a UF com maior rendimento mensal domiciliar *per capita* do Brasil (R$ 2.913,00).^10^ A despeito disso, persistem as desigualdades socioeconômicas na capital do país. Em 2020, o Índice de Vulnerabilidade Social do Distrito Federal (IVS-DF) foi de 0,34 em uma escala que varia de 0 a 1, na qual valores maiores representam maior vulnerabilidade social. Embora o IVS-DF seja considerado médio, há grandes disparidades entre as regiões administrativas, com a seguinte distribuição: muito baixo (14 regiões administrativas), baixo (11), médio (3), alto (1) e muito alto (3).^11^ Esse padrão assimétrico pode afetar significativamente a prevalência de gastos catastróficos no Distrito Federal. Adicionalmente, o Distrito Federal possui algumas particularidades em relação às demais UFs, como dupla atuação, englobando funções de estado e de município, e gestão centralizada, dada a ausência de instâncias municipais autônomas.^12^

O monitoramento da prevalência de gastos catastróficos em saúde pressupõe o acompanhamento desse indicador a curto e longo prazos. A falta de padronização nas estimativas pontuais compromete esse monitoramento, dificultando assim a atuação dos formuladores de políticas no seu combate. Foi identificado apenas um estudo que mensurou o gasto catastrófico em saúde do Distrito Federal, sendo somente para o ano de 2018. Avanços ainda são necessários para padronizar os métodos, estratégias empíricas e fontes de dados, a fim de se permitir o monitoramento mais acurado da proteção financeira em saúde, ainda mais em nível subnacional. O presente estudo traz, como principais contribuições, a uniformidade metodológica, empírica e de fonte de dados ao longo de diferentes períodos, além da produção de estimativas mais comparáveis em termos de evolução da prevalência de gastos catastróficos.

Este estudo tem por objetivo investigar a evolução da prevalência de gastos catastróficos no Distrito Federal em três momentos distintos (2003, 2009 e 2018), bem como identificar a composição dos gastos diretos com saúde nas famílias, nos respectivos anos. 

## MÉTODOS

### Desenho do estudo 

Trata-se de um estudo de série temporal, com abordagem descritiva, usando dados secundários da Pesquisa sobre Orçamentos Familiares (POF), contemplando os períodos de 2002/2003, 2008/2009 e 2017/2018. Os dados foram acessados via *website* do Instituto Brasileiro de Geografia e Estatística (IBGE), efetuando-se o *download* dos arquivos em 7 de março de 2023. Assim, buscou-se verificar eventuais desigualdades relacionadas à prevalência dos gastos catastróficos por recorte de consumo. 

### Contexto do estudo 

O Distrito Federal é a capital do Brasil, com área territorial de 5.760,784 km^2^, população residente de 2.817.068 pessoas e densidade demográfica de 489,01 habitantes por km^2^. O sistema de saúde do Distrito Federal possui uma organização complexa em comparação às outras UFs do Brasil, com entidades de diferentes personalidades jurídicas, finalidades e entregas à população.^13^ Além disso, o Distrito Federal contém o fundo constitucional, repasse da União que contempla vários setores, incluindo a saúde, por sediar a capital do país. Até há algum tempo, esse valor supria as contas da saúde, mas, com o passar dos anos, e em virtude da rápida transição demográfica, os recursos repassados tornaram-se insuficientes para manter a regularidade nas contas.^12^

### Participantes

Foram usados dados das três últimas edições da POF (2002/2003, 2008/2009 e 2017/2018). A POF é uma pesquisa realizada pelo IBGE com foco nas estruturas de consumo das famílias, possibilitando delinear um perfil das condições de vida da população com base na investigação do orçamento doméstico. A unidade de análise da POF é o domicílio, selecionado por meio de amostragem probabilística, com representatividade nacional, grandes regiões e UFs.^14^ Para cada edição da POF, foram utilizados os pesos amostrais da pesquisa. A POF contempla vários questionários, chamados de *cadernetas*. Para gerar os resultados deste estudo, foram extraídos dados das seguintes cadernetas, nas três versões da pesquisa: i) despesa individual, ii) morador, iii) condições de vida, iv) caderneta coletiva, v) domicílio, vi) outros rendimentos e vii) inventário. Para as POFs 2002-2003 e 2008-2009, também foram usados dados das cadernetas referentes à despesa de 90 dias, serviços domésticos e despesa de 12 meses; para as POFs 2008-2009 e 2017-2018, aluguel estimado; e para a POF 2017-2018, despesa coletiva e restrição-saúde. Como a POF inclui todos os membros da família, não há recorte de idade como critério de exclusão. No entanto, o chefe de família é adulto (idade maior ou igual a 18 anos).

### Variáveis e fontes de dados

A variável de interesse foi o gasto catastrófico com saúde das famílias, mensurado por meio dos gastos diretos em saúde (numerador) que excedam um determinado percentual da renda total ou consumo total familiar (denominador). Os percentuais mais utilizados na literatura são 10%, 25% e 40%,^1,15^ os quais também foram adotados neste estudo. Optou-se pelo recorte do consumo, por este ser mais sensível à capacidade de pagamento direto por cuidados de saúde, particularmente quando se deduzem gastos com necessidades básicas, como moradia e alimentação.^16^ A equação é expressa pela divisão dos gastos diretos em saúde (numerador) pelos gastos com consumo familiar (denominador), multiplicada por 100, para se obter o percentual. Para os limiares de 10% e 25%, o consumo total das famílias foi considerado o denominador da equação; por sua vez, no limiar de 40%, foram subtraídos do consumo total das famílias os gastos com aluguel e alimentação.

A composição dos gastos diretos em saúde (numerador) incluiu medicamentos e produtos de saúde, hospitalizações, consultas e exames, excluindo os pagamentos voluntários com planos e seguros de saúde. Essa é uma recomendação da Organização Mundial da Saúde e do Banco Mundial, segundo a qual qualquer reembolso proveniente de uma terceira parte (sistema público ou privado) deve ser excluído.^16^

Para verificação dos medicamentos com maior impacto nos gastos dos domicílios, eles foram classificados de acordo com o sistema de classificação anatômica terapêutica química (*anatomical therapeutic chemical* – ATC), em que as substâncias ativas são divididas em diferentes grupos, de acordo com o órgão ou sistema sobre o qual atuam e suas propriedades terapêuticas, farmacológicas e químicas.^17^ Adicionalmente, foi calculado o percentual de domicílios que tiveram restrição para a aquisição de medicamentos, com dados da caderneta restrição-saúde, disponível apenas para a POF 2017-2018. 

### Análise estatística

Os dados foram analisados de forma descritiva. Para tal, foi calculado o percentual de domicílios que incorreram em gastos catastróficos em saúde em três pontos do tempo (2003, 2009 e 2018), levando-se em consideração diferentes limiares (10%, 25% e 40%). Para a análise das desigualdades socioeconômicas nos gastos catastróficos em saúde, os domicílios foram divididos em cinco partes iguais (quintis), estratificados por renda e por consumo. Foram considerados os pesos amostrais das três POFs. As análises foram feitas utilizando-se o Stata 14.0. 

Os valores em reais (R$) para a comparação entre os anos foram corrigidos conforme o Índice Nacional de Preços ao Consumidor Amplo, tendo sido os valores ajustados a preços de agosto de 2023,^18^ último mês disponível à época da realização do estudo. Para a conversão dos valores das POFs para os valores atuais, foi utilizada a data referencial das pesquisas, disponibilizadas pelo IBGE. O mês de referência para a realização da conversão dos valores foi o mês de janeiro do último ano de cada POF (2003, 2009 e 2018).^14,19,20^ Ademais, foi conduzida uma análise descritiva da composição dos gastos diretos em saúde por finalidade, incluindo gastos com medicamentos e produtos de saúde, hospitalizações, consultas e exames, e planos de saúde. Destaque foi dado aos gastos diretos com medicamentos, adicionando-se dados de dispêndio por classificação dos medicamentos autorrelatados.

### Aspectos éticos 

Por se tratar de uma análise com base em fontes de pesquisa secundária, o estudo não necessitou passar por Comitê de Ética e Pesquisa, conforme Resolução nº 466 do Conselho Nacional de Saúde. 

## RESULTADOS

Na composição da POF, foram selecionados 1.214 domicílios em 2002/2003, 1.703 domicílios em 2008/2009 e 1.850 domicílios em 2017/2018, para a realização da pesquisa no Distrito Federal. Em relação aos domicílios que foram entrevistados na pesquisa, em 2002/2003 houve a entrevista de 981 domicílios (80,8% dos domicílios selecionados); em 2008/2009, foram 977 domicílios (57,3% dos domicílios selecionados); e em 2017/2018, 1.331 domicílios (71,9% dos domicílios selecionados). Na composição de domicílios analisados, após o processo de exclusão devido à ausência de dados, a amostra selecionada para o estudo ficou em 754 domicílios analisados na POF 2002/2003 (76,8% dos domicílios entrevistados), 695 domicílios analisados na POF 2008/2009 (71,1% dos domicílios entrevistados) e 1.000 domicílios na POF 2017/2018 (75,1% dos domicílios entrevistados). Utilizando-se os pesos amostrais, a representatividade de habitantes do Distrito Federal, nos anos estudados, foi de 1.713.092 habitantes na POF 2002/2003, 1.880.005 habitantes na POF 2008/2009 e 2.316.798 habitantes na POF 2017/2018 (Figura 1).

**Figura 1:** Fluxograma dos domicílios incluídos na análise de três pesquisas representativas nacionais sobre consumo domiciliar, 2002/2003, 2008/2009 e 2017/2018, Distrito Federal

Pelas três formas de cálculo da prevalência dos gastos catastróficos, verificou-se aumento da prevalência entre 2003 e 2009, seguida por uma queda entre 2009 e 2018. No entanto, ao se considerar 2003 como período base, identificou-se crescimento da prevalência de gastos catastróficos em relação a 2018, com exceção da POF 2017/2018, considerando-se o limiar de 40% ou mais do consumo total, menos alimentação e aluguel (2,7% [IC_95_% 1,4;3,8] *versus* 2,5% [IC_95_% 1,5;3,4], respectivamente) (Tabela 1). 

**Tabela 1:** Prevalência dos gastos catastróficos em (%) com intervalos de confiança de 95% (IC_95_%), em saúde por quintil do consumo para limiares selecionados (10%, 25% e 40%) do consumo, estratificado por ano da Pesquisa de Orçamentos Familiares, Distrito Federal

Quintil do consumo	Famílias com comprometimento de 10% ou mais do consumo total			Famílias com comprometimento de 25% ou mais do consumo total			Famílias com comprometimento de 40% ou mais do consumo total menos alimentação e aluguel		
	POF ^a^ 2002/2003 % (IC _95_ % )	POF 2008/2009 % (IC _95_ % )	POF 2017/2018 % (IC _95_ % )	POF ^a^ 2002/2003 % (IC _95_ % )	POF 2008/2009 % (IC _95_ % )	POF 2017/2018 % (IC _95_ % )	POF ^a^ 2002/2003 % (IC _95_ % )	POF 2008/2009 % (IC _95_ % )	POF 2017/2018 % (IC _95_ % )
1	12,5 (6,3;18,6)	12,1 (5,9;18,1)	21,4 (15,5;27,2)	3,9 (0,2;7,5)	2,4 (-0,6;5,4)	4,2 (1,3;7,1)	5,3 (1,4;9,2)	4,8 (0,8;8,6)	5,5 (2,2;8,7)
2	11,6 (6,2;17,0)	14,0 (7,3;20,6)	16,7 (11,2;22,2)	1,5 (-0,1;3,1)	4,5 (0,1;8,9)	2,0 (0,1;3,9)	2,1 (0,3;3,9)	6,3 (1,2;11,3)	2,0 (-0,1;4,0)
3	11,3 (5,6;17,0)	13,4 (7,0;19,6)	11,7 (7,1;16,2)	1,6 (-0,4;3,6)	3,3 (0,1;6,44)	2,3 (-0,1;4,6)	1,5 (-0,5;3,4)	3,2 (0,1;6,3)	1,2 (-0,3;2,7)
4	13,3 (6,7;19,8)	19,3 (11,7;26,7)	12,3 (7,5;17,0)	1,4 (-0,6;3,3)	5,9 (1,5;10,2)	1,7 (-0,1;3,3)	2,5 (0,1;4,9)	4,8 (0,7;8,8)	2,2 (0,2;4,1)
5	12,8 (6,8;18,6)	15,8 (9,06;22,54)	10,3 (6,2;14,2)	2,5 (-0,2;5,1)	2,6 (-0,4;5,5)	3,3 (0,9;5,6)	1,7 (-0,6;4,0)	2,0 (-0,8;4,8)	2,1 (0,2;3,9)
DF	12,3 (9,6;14,9)	15,3 (12,1;18,3)	14,1 (11,8;16,2)	2,1 (1,0;3,2)	3,8 (2,1;5,5)	2,7 (1,6;3,7)	2,7 (1,4;3,8)	4,1 (2,3;5,8)	2,5 (1,5;3,4)

a) Pesquisa de Orçamentos Familiares.

Considerando-se a distribuição do consumo dos domicílios e o limiar de 10% ou mais do consumo como indicador de gasto catastrófico, verificou-se que os domicílios de menor consumo (quintil 1) tiveram um aumento de 71,2% na prevalência dos gastos catastróficos entre 2003 e 2018, enquanto os domicílios de maior consumo (quintil 5) apresentaram uma diminuição de 19,5% na prevalência no mesmo período. Por seu turno, nos limiares de 25% e 40%, os dois extremos da cauda da distribuição (quintis 1 e 5) apresentaram crescimento da prevalência de gastos catastróficos, sendo esse crescimento mais pronunciado nos domicílios de maior consumo (limiar de 25%: quintil 1 = 7,6% *versus* quintil 5 = 32,0%; limiar de 40%: quintil 1 = 3,7% *versus* quintil 5 = 23,5%) (Tabela 1). 

A Tabela 2 mostrou que a composição dos gastos diretos em saúde diferiu em relação à renda dos domicílios, observando-se predomínio dos gastos com medicamentos nos domicílios de menor renda (quintil 1); e com planos de saúde, nos domicílios de maior renda (quintil 5). Ademais, os gastos com hospitalização foram próximos de zero em todos os quintis de renda. Não foi encontrado um padrão bem definido da porcentagem dos gastos com consultas e exames em relação aos quintis de renda, embora as duas caudas da distribuição tenham porcentagens bem distintas (quintil 1 = 5,3% *versus* quintil 5 = 16,6% na POF 2017/2018). 

**Tabela 2:** Distribuição (%) com intervalos de confiança de 95% (IC95%), do gasto direto em saúde por quintil de renda, estratificado por tipo de despesa e pelo ano da Pesquisa de Orçamentos Familiares, Distrito Federal

Tipo de gasto direto em saúde	Ano da POF ^b^	Quintil da renda					
		1 (↓ renda) % (IC _95_ % )	2 % (IC _95_ % )	3 % (IC _95_ % )	4 % (IC _95_ % )	5 (↑ renda) % (IC _95_ % )	DF ^a^ % (IC _95_ % )
Medicamento	2002/2003	86,5 (80,7;92,3)	71,4 (63,7;78,9)	57,3 (49,5;65,0)	42,7 (36,1;49,2)	26,4 (21,0;31,7)	56,8 (53,4;60,1)
	2008/2009	83,4 (76,7;90,0)	79,2 (71,6;86,7)	82,8 (76,6;88,9)	65,6 (57,5;73,6)	60,1 (52,8;67,3)	72,3 (68,8;75,7)
	2017/2018	88,3 (81,5;95,0)	77,9 (70,7;84,9)	73,5 (66,8;80,1)	66,0 (59,9;72,0)	41,7 (38,4;44,8)	54,7 (52,1;57,3)
Hospitalização	2002/2003	- (-)	- (-)	- (-)	- (-)	0,4 (-0,3;1,0)	0,1 (-0,1;0,2)
	2008/2009	- (-)	- (-)	- (-)	- (-)	- (-)	- (-)
	2017/2018	- (-)	- (-)	- (-)	- (-)	0,1 (-0,1;0,2)	0,1 (-0,1;0,2)
Consulta e exame	2002/2003	12,2 (6,7;17,7)	25,2 (17,7;32,6)	21,0 (14,8;27,0)	27,3 (21,2;33,3)	28,7 (22,3;34,9)	23,0 (20,1;25,9)
	2008/2009	12,1 (6,1;18,1)	16,1 (9,3;22,7)	10,7 (6,0;15,2)	14,7 (9,6;19,8)	15,7 (10,2;21,2)	14,1 (11,5;16,6)
	2017/2018	5,3 (1,4;9,1)	17,4 (10,9;23,7)	16,9 (11,5;22,1)	18,4 (13,8;22,9)	16,6 (13,5;19,7)	16,4 (14,2;18,5)
Plano de saúde privado	2002/2003	1,2 (0,1;2,3)	3,4 (0,5;6,2)	21,7 (14,2;29,1)	30,0 (23,2;36,7)	44,6 (38,2;50,9)	20,1 (17,5;22,7)
	2008/2009	4,5 (1,1;7,8)	4,7 (1,2;8,3)	6,5 (2,2;10,8)	19,7 (11,9;27,4)	24,1 (17,6;30,5)	13,6 (10,8;16,4)
	2017/2018	6,4 (1,2;11,5)	4,8 (1,0;8,5)	9,7 (4,6;14,7)	15,6 (10,5;20,6)	41,6 (37,9;45,3)	28,8 (26,1;31,5)

a) Distrito Federal; b) Pesquisa de Orçamentos Familiares.

Em relação aos valores médios despendidos com medicamentos nos domicílios do Distrito Federal, corrigidos pela inflação do período, verificou-se um aumento dos gastos entre 2003 e 2009 (45,0%), seguido de uma pequena queda entre 2009 e 2018 (-0,5%). Observou-se padrão semelhante ao serem consideradas as caudas extremas da distribuição de renda, tendo o crescimento dos gastos sido mais acentuado nos domicílios mais pobres, entre 2003 e 2009 (quintil 1 = 67,3% versus quintil 5 = 30,5%), e maior a queda dos gastos nos domicílios mais ricos (quintil 1 = -9,2% versus quintil = -14,4%), entre 2009 e 2018 (Tabela 3).

**Tabela 3:** Distribuição do gasto direto com medicamentos por quintil de renda, estratificado pela Classificação Anatômica-Terapêutico-Química (ATC) e pelo ano da Pesquisa de Orçamentos Familiares, Distrito Federal

Classificação ATC ^a^	Ano da POF ^c^	Quintil da renda R$)					
		1 (↓ renda)	2	3	4	5 (↑ renda)	DF ^b^
Aparelho digestivo e metabolismo (A)	2002/2003	1,90	5,29	8,72	25,73	25,18	12,80
	2008/2009	23,67	28,07	22,73	16,45	54,03	30,53
	2017/2018	11,13	15,28	25,10	28,47	54,67	29,19
Aparelho cardiovascular (C)	2002/2003	4,53	5,14	11,56	51,03	50,55	23,39
	2008/2009	17,11	20,12	50,89	56,62	93,89	51,93
	2017/2018	9,36	21,37	25,97	39,70	43,29	29,53
Medicamentos dermatológicos (D)	2002/2003	2,31	5,06	4,30	4,00	12,17	5,35
	2008/2009	0,40	1,31	0,06	0,56	9,35	2,83
	2017/2018	0,45	3,74	1,29	6,27	13,04	5,62
Aparelho geniturinário e hormônios sexuais (G)	2002/2003	2,68	7,02	1,83	1,94	7,95	4,26
	2008/2009	1,54	3,13	6,50	10,25	3,62	5,14
	2017/2018	1,30	4,18	4,67	6,90	3,38	4,12
Anti-infecciosos gerais para uso sistêmico (J)	2002/2003	2,20	11,39	20,52	22,06	15,06	13,92
	2008/2009	2,69	3,17	2,27	8,39	8,46	5,40
	2017/2018	4,52	6,85	4,77	14,54	12,89	9,19
Agentes antineoplásicos e imunomoduladores (L)	2002/2003	9,17	9,52	19,75	17,01	28,94	16,24
	2008/2009	5,51	18,61	7,59	16,68	33,63	17,91
	2017/2018	3,56	4,70	14,90	9,86	11,77	9,26
Sistema nervoso (N)	2002/2003	4,59	3,49	3,54	21,76	25,72	11,30
	2008/2009	3,35	5,01	27,54	12,83	31,04	17,18
	2017/2018	7,57	25,19	16,91	52,53	24,69	26,11
Produtos antiparasitários (P)	2002/2003	0,49	0,47	0,01	-	0,13	0,23
	2008/2009	0,07	0,01	-	0,26	-	0,07
	2017/2018	0,38	1,09	-	0,39	0,41	0,39
Aparelho respiratório (R)	2002/2003	4,00	6,37	10,24	12,94	9,57	8,47
	2008/2009	2,63	12,37	13,05	13,06	16,00	11,99
	2017/2018	5,95	6,76	8,99	11,54	14,97	10,12
Órgãos dos sentidos (S)	2002/2003	0,36	0,48	7,08	5,51	14,30	5,10
	2008/2009	0,03	0,30	-	1,51	1,01	0,64
	2017/2018	2,65	2,72	3,88	5,49	9,62	5,26
Vários (V)	2002/2003	12,20	10,20	19,79	20,40	31,87	18,24
	2008/2009	17,34	17,44	19,93	46,59	37,99	29,39
	2017/2018	20,57	35,69	42,43	51,15	58,51	43,34
Total Médio	2002/2003	44,43	64,43	107,34	182,38	221,44	119,30
	2008/2009	74,34	109,54	150,56	183,20	289,02	173,01
	2017/2018	67,44	127,57	148,91	226,84	247,24	172,13

a) Classificação Anatômica-Terapêutico-Química; b) Distrito Federal; c) Pesquisa de Orçamentos Familiares.

Na Tabela 3, também é possível verificar quais medicamentos absorveram maior proporção dos gastos diretos com essa finalidade nos domicílios do Distrito Federal. Houve predomínio dos medicamentos relacionados às classificações V (vários), C (aparelho cardiovascular) e A (aparelho digestivo e metabolismo), representando 45,6% do total de gastos com medicamentos em 2003, 64,6% em 2009, e 59,2% em 2018. Não foram identificadas alterações substanciais na composição dos medicamentos por recorte de renda.

A Tabela 4 mostra que as famílias de baixa renda tiveram maior restrição na aquisição de medicamentos em todas as classificações da ATC. Os medicamentos relacionados ao sistema nervoso (N) foram a classificação com menor diferença entre os extremos da renda, sendo o percentual de restrição 1,5 vez maior no quintil 1 do que no quintil 5 (2,2% *versus* 1,4%, respectivamente). Já a classificação do aparelho digestivo e metabolismo (A) apresentou a maior diferença no percentual de restrição, sendo 11,5 vezes maior no quintil 1 em comparação com o quintil 5.

**Tabela 4:** Percentual dos domicílios que tiveram restrição para a aquisição de medicamentos por quintil de renda, estratificado pela Classificação Anatômica-Terapêutico-Química (ATC), Distrito Federal, POF 2017/2018

Tipo de medicamento que houve restrição (classificação ATC ^a^ )	Ano da POF ^c^	Quintil da renda em %					
		1 (↓ renda)	2	3	4	5 (↑ renda)	DF ^b^
Aparelho digestivo e metabolismo (A)	2017/2018	4,6	0,9	1,7	3,6	0,4	2,1
Aparelho cardiovascular (C)	2017/2018	5,3	4,4	4,6	5,6	2,1	4,2
Medicamentos dermatológicos (D)	2017/2018	1,8	-	0,9	1,2	1,0	1,0
Aparelho geniturinário e hormônios sexuais (G)	2017/2018	0,4	0,6	-	0,7	0,2	0,4
Anti-infecciosos gerais para uso sistêmico (J)	2017/2018	1,7	-	1,2	0,5	0,2	0,7
Agentes antineoplásticos e imunomoduladores (L)	2017/2018	2,7	1,3	0,8	0,7	1,3	1,3
Sistema nervoso (N)	2017/2018	2,2	1,3	1,3	1,5	1,4	1,5
Produtos antiparasitários (P)	2017/2018	0,4	-	-	-	-	-
Aparelho respiratório (R)	2017/2018	3,2	0,3	2,0	-	-	1,0
Órgãos dos sentidos (S)	2017/2018	0,7	0,4	-	0,3	0,3	0,3
Vários (V)	2017/2018	12,5	10,7	6,8	6,0	1,8	7,1

a) Classificação Anatômica-Terapêutico-Química; b) Distrito Federal; c) Pesquisa de Orçamentos Familiares.

## DISCUSSÃO

Neste estudo, foi possível monitorar a prevalência dos gastos catastróficos no Distrito Federal em três pontos do tempo. De modo geral, o pico da prevalência dos gastos catastróficos ocorreu em 2009, independentemente da forma de mensurá-la (limiar de 10%, 25% ou 40%). Ademais, os domicílios com menor consumo (quintil 1) tendem a gastar proporcionalmente mais com cuidados de saúde do que os domicílios com maior consumo (quintil 5). Em outros termos, domicílios com menor renda tendem a ter maior prevalência de gastos catastróficos em saúde. Cabe ressaltar ainda que os medicamentos possuem um grande peso no gasto direto em saúde das famílias de baixa renda, representando mais de 83% dessas despesas. Os medicamentos que demandaram maior desembolso das famílias foram aqueles relacionados ao aparelho digestivo e metabolismo (A), aparelho cardiovascular (C) e vários (V). Essas classificações também foram as que geraram maior restrição para sua aquisição. 

Ao se considerar o limiar de 10% do consumo, a prevalência dos gastos catastróficos em saúde oscilou entre 12,3% (2003), 15,3% (2009) e 14,1% (2018) dos domicílios do Distrito Federal. Essas estimativas foram menores do que aquelas encontradas por Araújo e Coelho (2021) para o Distrito Federal (28,7%).^7^ Embora os dois estudos tenham usado a mesma base de dados (POF 2017/2018), a adoção de diferentes estratégias metodológicas pode explicar essas diferenças. No estudo de Araújo e Coelho (2021),^7^ realizando estimativa sobre o Brasil e as UFs, não há relato sobre o uso do peso amostral da pesquisa, a forma como o consumo foi calculado (denominador) e o número de domicílios incluídos no cálculo. Outros estudos forneceram estimativas da prevalência de gastos catastróficos em saúde em âmbito nacional (tendo o Brasil em sua análise), cobrindo os períodos de 1987 a 2003, 2002 e 2003, e 2002 a 2009, porém, não estratificados por UF.²¹^,^²²^,^²³

Por sua vez, em nível subnacional, foram encontrados dois estudos, um contemplando o estado de Minas Gerais,^24^ com estimativas entre os anos de 2009 e 2013, e outro o município de Porto Alegre,^25^ em que a pesquisa utilizou dados entre julho e setembro de 2003. No município de Porto Alegre,^25^ foi realizado um estudo utilizando dados com amostra representativa de famílias atendidas pela Estratégia Saúde da Família entre julho e setembro de 2003. Foram utilizados os limiares de 5%, 10% e 20% da renda familiar e de 40% da capacidade de pagar do domicílio, para se calcular a proporção de domicílios que incorriam em gastos catastróficos em Porto Alegre. Para o limiar de 10%, foi encontrada uma prevalência de 28,8% das famílias. Além de serem UFs distintas, também há diferenças metodológicas entre os estudos – por exemplo, a definição do denominador do limiar, que, no nosso estudo, foi o consumo em vez da renda.^25^ No estado de Minas Gerais,^24^ foi avaliado o gasto catastrófico em saúde entre os anos de 2009, 2011 e 2013, utilizando a Pesquisa por Amostra de Domicílios de Minas Gerais. Para mensurar a prevalência do gasto catastrófico em saúde, os limiares de 10% e 25% da renda domiciliar foram utilizados. No limiar de 10%, de 2009 para 2011, houve um aumento de 22,5% na proporção de gastos catastróficos, e de 2011 para 2013, uma diminuição de 13,5%. No limiar de 25%, de 2009 para 2011, ocorreu um aumento de 20,3%, e uma diminuição de 24,4% de 2011 para 2013. Esse mesmo padrão foi observado no nosso estudo, nos limiares de 10%, 25% e 40% na prevalência de gasto catastrófico pelo consumo.^24^

A relação inversa entre renda/consumo e gasto catastrófico também foi observada em outras publicações brasileiras, todas contemplando o Brasil em sua análise, com períodos que vão de 1987 a 2009,^7,21,23^ corroborando os achados do nosso estudo. Em nível global, também foi encontrada essa relação inversa entre as variáveis em tela em Bangladesh, no ano de 2010,^26^ na Correia do Sul, entre os anos de 2009 e 2012^27,^ e em países-membros da União Europeia, no período de 2000 a 2017.^5^

Os gastos diretos com medicamentos como componente de maior peso para os domicílios mais pobres também foram observados em outros estudos. Em nível nacional com base na POF 2017-2018^7^, os gastos com medicamentos foram maiores no grupo de famílias mais pobres (decil 1), representando 84% dos gastos de saúde desse grupo, diferentemente do grupo de pessoas mais ricas (decil 10), em que esse valor foi somente de 29%. Em Minas Gerais,^24^ os gastos com medicamentos, nos três anos analisados (2009, 2011 e 2013), representaram aproximadamente 94% do desembolso direto. No ano de 2003, em Porto Alegre,^25^ utilizando dados dos domicílios cobertos pelo Programa Saúde da Família, o principal gasto com saúde no grupo de domicílios mais pobres (quintil 1) foi com os medicamentos, em média R$ 20,07 (valor referente a 2003, e valor ajustado pela inflação, a preços de agosto de 2023, de R$ 45,10), enquanto no grupo de domicílios mais ricos o principal gasto foi com os planos de saúde, em média R$ 138,90 (valor referente a 2003, e valor ajustado pela inflação, a preços de agosto de 2023, de R$ 312,12). 

No SUS, a assistência farmacêutica tem apresentado avanços significativos em termos normativos, de que são exemplos a criação da Política Nacional de Medicamentos (1998), a Política Nacional de Assistência Farmacêutica (2004) e o Programa Farmácia Popular do Brasil (2004).^28^ A despeito desses avanços, ainda persistem problemas de acesso aos medicamentos, particularmente pelas pessoas de baixa renda, como mostrado em nosso estudo, em que mais de 83% do dispêndio em saúde das famílias se destinou à aquisição de medicamentos no quintil 1 (domicílios de mais baixa renda). Ao se desagregar o gasto com medicamentos por finalidade terapêutica, predominam as classificações que incluem a diabetes (A) e a hipertensão (C), sendo esses medicamentos parcialmente dispensados na atenção primária. Segundo dados na Pesquisa Nacional sobre Acesso, Utilização e Promoção do Uso Racional de Medicamentos (PNAUM), 25,7% (IC_95_% 23,4;28,2) e 21,1% (IC_95_% 18,1;24,4) dos medicamentos utilizados para tratar a hipertensão^29^ e a diabetes^30^ foram pagos pelas famílias (gasto direto), respectivamente.

Este estudo contribui com a literatura ao sistematizar evidências sobre a prevalência dos gastos catastróficos no Distrito Federal, contemplado dados de três mensurações, no período de 15 anos. Ademais, fornece dados inéditos sobre a decomposição dos gastos com medicamentos por finalidade terapêutica e sinaliza a ocorrência de restrição à aquisição de medicamentos por quintil de renda. Ressalta-se que a fonte de dados usada neste estudo está alinhada com as recomendações internacionais sobre o tema,^1^ incluindo amostragem probabilística e dados detalhados sobre consumo e renda. Por fim, os resultados deste estudo permitem monitorar a (des)proteção financeira na saúde frente aos gastos catastróficos, em nível subnacional. 

A despeito das fortalezas do estudo, algumas limitações devem ser mencionadas. Primeiro, os dados públicos da POF não permitem desagregar os microdados por região administrativa do Distrito Federal, o que limitou nossa análise sobre as disparidades existentes entre as regiões administrativas (aspecto geográfico). Para contornar esse fato, foram conduzidas análises por quintis de renda, no sentido de captar as assimetrias na prevalência dos gastos catastróficos por recorte socioeconômico. De fato, as pessoas com menor consumo e/ou menor renda tendem a ser mais afetadas pelos gastos catastróficos e pela restrição na compra de medicamentos. Segundo, ao longo das três últimas edições da POF, ocorreram modificações em alguns itens de consumo relacionados à saúde; porém, essas alterações foram limitadas a poucos itens, com impacto reduzido na comparabilidade das pesquisas. Terceiro, pelo fato de a amostra da POF para o Distrito Federal ser relativamente pequena, com dados faltantes (*missing data*) e a prevalência de gastos catastróficos ser pequena (especialmente nos limiares de 25% e 40%), não foi possível estimar os fatores associados aos gastos catastróficos. Como consequência, poder-se-ia apresentar relações entre variáveis não significativas de forma espúria. O número pequeno de observações também aumenta a incerteza sobre o real valor da prevalência dos gastos catastróficos no Distrito Federal. Para contornar essa situação, foram incluídos os intervalos de confiança. 

Por fim, conclui-se que houve aumento da desproteção financeira em saúde das famílias do Distrito Federal. A prevalência de gastos catastróficos na saúde aumentou no Distrito Federal, evoluindo em 1,8 ponto percentual (p.p.) de 2003 para 2018, atingindo seu pico em 2009 (aumentando 3 p.p. de 2003 para 2009), considerando-se a ocorrência do gasto catastrófico como dispêndios iguais ou superiores a 10% do consumo dos domicílios. Como recomendação para os serviços de saúde, há espaço para ampliar sua atuação com foco na equidade, principalmente em relação ao acesso a medicamentos para doenças como diabetes e hipertensão. Isto pode contribuir efetivamente para aumentar a proteção financeira em saúde das famílias com menor renda, dado que a aquisição de medicamentos é o maior componente de gasto direto dessas famílias.
